# Study of the Association between Human T-cell Lymphotropic Virus Type 1 Infection and Lichen Planus

**Published:** 2013-03

**Authors:** Mahnaz Banihashemi, Bita Kiafar, Mohammad Ashkezari, Noorieh sharifi, Nasibe Pishgooei, Fatemah Livani, Reyhaneh Bazargani, Mohammad Esmail Khayami, Mohammad Javad Yazdanpanah

**Affiliations:** 1 Research Center for Skin Diseases and Cutaneous Leishmaniasis, Faculty of medicine, Mashhad University of Medical Sciences, Mashhad, Iran; 2 Department of Pathology, Faculty of medicine, Mashhad University of Medical Sciences, Mashhad, Iran; 3 Research Center of Iranian Blood Transfusion Organisation, Khorasan Razavi, Mashhad, Iran

**Keywords:** Human T-cell Lymphotropic Virus Type 1, Lichen Planus, Skin disease

## Abstract

***Objective(s):*** Lichen Planus is a common disease with unknown etiology which affects the skin and mucosa. Recent studies have focused on the possible role of the virus in the pathogenesis of Lichen Planus. The purpose of this study was to determine the association between the human T-cell lymphotropic virus type 1 and Lichen Planus.

***Materials and Methods:*** This case control study was conducted on a total of 200 patients. The case group consisted of 100 patients with a confirmed histopathological diagnosis of lichen planus disease, and the control group consisted of 100 healthy blood donors without any signs or symptoms of skin diseases, and who were similar in age and sex to the case group. Blood samples of both participants in the case and control groups were examined for the presence of anti –HTLV-I antibodies using the ELISA method. The polymerase chain reaction for human T-cell lymphotropic virus type 1 was conducted in cases in which the findings for antihuman T-cell lymphotropic virus type 1 antibody test was positive, and statistical analysis was conducted on the obtain results.

***Results:*** One case in the case group was infected with human T-cell lymphotropic virus type 1; however, no infection was observed in the control group. The difference was not statistically significant (P = 1).

***Conclusion: ***Based on the obtained results, no association was observed between the human T-cell lymphotropic virus type 1 infection and Lichen Planus.

## Introduction

Lichen Planus (LP) is a common disease with unknown etiology which affects the skin and mucous membrane. The pathogenesis of LP is an immune disease, and increasing evidences based on immune function of the T cell indicates that it causes basal keratinocytes damages. Numerous clinical and case examples exist that introduce a probable association between the number of exogenous factors, such as viruses, drugs and contact allergens and the genetics of LP (1).

Among the potential exogenous factors, lately, attention has been given to the role of viruses in the pathogenesis of LP. The hepatitis C virus (HCV) as a factor of LP was introduced, although there has been some controversies of epidemiologic evidence to support this association (2).

Based on our clinical observations, several patients had lichen planus and the human T-cell lymphotropic virus type 1 (HTLV-I) infection coexistence. Therefore, we evaluated the role of HTLV-I infection as a possible factor in the pathogenesis of patients with LP disease.

## Materials and Methods

This was a case-control study which was conducted in the Dermatology Clinic of Ghaem Hospital from March 2009 to September 2010. One hundred patients were selected as the case group after clinical diagnosis and the histological confirmation of LP (49 males and 51 females). One hundred healthy blood donors without any signs and symptoms of skin disease, who were identical as the case group regarding age and sex were entered the study as the control group. The sampling method was simple nonrandom (sequential).

The project was approved by the regional ethic committee, and a written consent was taken from all patients .The diagnosis was based on a clinical diagnosis and confirmed by a biopsy of the skin lesions. The histopathological characteristics that were evaluated as the diagnostic criteria included hyperkeratosis, local thickening of the granular cell layer, a band like lymphocytic infiltration at the dermo-epidermal junction, and localized signs of basal cell degeneration. The exclusion criteria were unwillingness of the patient to participate in the study, being suspected of having a rash caused by a lichenoid drug eruption, or being under 18 years old. By using the anti-HTLV-I method, the patients underwent the third generation enzyme-linked immunosorbent assay (ELISA III Dia pro Diagnostic Bioprobes Crop, Milano, Italy). To confirm the diagnosis, seropositive patients were evaluated for RNA and HTLV-I by the polymerase chain reaction method (3). Statistical analysis was performed by the SPSS 11.5 software. The student t-test was used to compare quantitative variables, and the X2 test for qualitative parameters. The significance level for all the tests was 0.05.

**Table 1:** The Frequency of HTLV-I Infection Among the Lichen Planus Cases and Control Group

Group HTLV – I Infection	Case		Control	
	Percent	Number	Percent	Number
Positive	1	1	0	0
Negative	99	99	100	100
Total	100	100	100	100

There was not a significant difference between the case and control groups by using the exact Fisher test (P= 1)

## Results

The age of the patients was 18 to 65 years and the mean age for the case group was 38.9 ± 13.4 years and for the control group was 38.1 ± 13.3 years. There was only one case of HTLV-I seropositive by ELISA method, among all 100 patients with LP which was confirmed by the PCR method. The results are shown in Table 1.

## Discussion

Despite advances made in understanding the immunopathogenesis of LP, the primary initiating factor or factors is not well known yet. Clinical evidence has shown the relationship between exposure to factors such as viruses, drugs and contact allergens with the manifestation of LP (1). 

Considering the possible role of viruses in the development of LP, in researches on transmitted viruses through transfusion results could not show the direct role of this factor in the development of LP (4). By in situ hybridization methods and immunohistochemistry techniques, human herpes virus-6 was found in 67-100% of oral LP lesions; whereas, in the normal oral tissue samples, results were negative (1). In some small studies, DNA of herpes virus 1, cytomegalovirus and human herpes virus 6 has been found occasionally in oral LP, mainly in the erosive lesions (5-7).

In a retrospective study on tissue samples of LP lesions, DNA of the human herpes virus 7 (HHV-7) was discovered in 11 of 18 samples (8). By using the nested polymerase chain reaction, the Epstein-Barr virus (EBV) DNA has appeared in 0% to 50% of oral LP samples. However, it is not clear whether EBV had any role in the pathogenesis of the disease or was added secondary to oral LP lesions (9).

There are a few numbers of reports indicating the manifestation of lichenoid lesions in patients with the human immunodeficiency virus (HIV), but most of them can be associated with the treatment of Zidovudine or Ketoconazole (10).

Also, there are a few reports indicating lichenoid rash following various types of the hepatitis B (HBV) vaccination (11). Studies on the detection of different types of HPV in oral LP were limited and include a few cases. The results were ambiguous and positive cases range from 0% to 87% (12-13). In fact, human papiloma DNA does not ecessarily prove any causal relationship and may be caused by the disease process or immunosuppressive treatment.

The city of Mashhad, which is the capital of Khorasan Razavi province in Iran, is an endemic disease region and the prevalence of the disease is 2.3% (14). According to the information, up to now no study on the association of HTLV– I infection and lichen planus has been performed.

Based on the results of this study, there was not any link between HTLV– I infection and LP.
